# Mechanism of continuous exercise intention after sport injury: a chained HBM-TPB mediation model

**DOI:** 10.3389/fpubh.2026.1894556

**Published:** 2026-09-07

**Authors:** Xuanming Hu, Peiyou Chen

**Affiliations:** School of Sports Science and Physical Education, Nanjing Normal University, Nanjing, Jiangsu, China

**Keywords:** chained mediation model, control beliefs regarding exercise rehabilitation, Health Belief Model, Theory of Planned Behavior, university students

## Abstract

**Background:**

Sports injuries are prevalent among university students, yet psychological mechanisms of post-injury exercise maintenance intention need further study. Integrating the Health Belief Model (HBM) and Theory of Planned Behavior (TPB), this study examined how risk perceptions and cognitive factors were associated with continuous exercise intention, focusing on the pivotal role of control beliefs regarding exercise rehabilitation in translating risk perception into volition.

**Methods:**

A cross-sectional survey sampled 421 university students who had a sports injury in the past 12 months. We integrated HBM and TPB, reconstructing perceived behavioral control and self-efficacy into control beliefs regarding exercise rehabilitation. SPSS 26.0 and AMOS 26.0 were used for SEM and bootstrapping mediation tests.

**Results:**

The model fit well (*χ^2^/df* = 2.799, CFI = 0.907, RMSEA = 0.065). Perceived susceptibility and health benefits were positively associated with attitude toward sustained exercise; perceived severity was negatively associated with attitude (*β* = −0.332, *p* < 0.001); perceived barriers had no significant direct effect. Mediation analyses revealed a dual-route pattern: perceived health benefits were associated with intention through the chain of attitude → control beliefs [95% CI (0.1135, 0.2500)]. Perceived susceptibility and barriers were indirectly related to lower control beliefs, and this relationship did not operate through attitude. Subjective norm was associated with intention, fully mediated by control beliefs (Effect = 0.3505). Control beliefs showed the largest predictive effect size on intention in the present model (*β* = 0.824, *p* < 0.001).

**Conclusion:**

Continuous post-injury exercise intention was associated with a stepwise path of attitude → control beliefs → intention. Perceived health benefits were associated with intention through this chain, while the pattern for perceived severity was consistent with fear-avoidance theory. Control beliefs connected internal values and social norms. The findings suggest that interventions may benefit from shifting from risk intimidation to benefit empowerment, and from reconstructing rehabilitation control through social support networks—though these candidate directions await experimental validation.

## 1 Introduction

For university students who have experienced sports injuries, overcoming the fear of reinjury to resume and sustain physical exercise represents a critical psychological challenge in their rehabilitation process. The practical urgency of this challenge stems from the high incidence of sports injuries within this demographic. For instance, a multi-center cross-sectional study (*n* = 5,341) targeting Chinese university students indicated that the incidence rate of experiencing at least one sports injury in the past 12 months was 24.2% (26.2% for males, 23.2% for females) (1). Among American collegiate athletes, this figure is equally significant, with injury incidence rates ranging from 68.45 to 75.65 per 1,000 athlete-exposures (2). Injuries not only cause immediate physical dysfunction and medical expenses but can also trigger long-term psychological sequelae—kinesiophobia, reinjury anxiety, and exercise avoidance behaviors (3, 4). A substantial proportion of injured students completely cease exercising during the rehabilitation period or even after full recovery, creating a vicious cycle of injury, detraining, physical deconditioning, and subsequent reinjury (5).

Unlike general exercise adherence, the decision to sustain exercise post-injury is embedded within a unique tension of risk–benefit assessment: individuals must simultaneously weigh the potential threat of reinjury against the functional desire to resume physical activity (6, 7). Among classic frameworks explaining such health behavioral intentions, the Health Belief Model (HBM) emphasizes the risk assessment of health threats, whereas the Theory of Planned Behavior (TPB) highlights the socio-cognitive drivers of intention—namely attitude, subjective norm, and perceived behavioral control. In recent years, scholars have widely advocated for the integration of the HBM’s risk perception pathways with the TPB’s socio-cognitive pathways to construct a more comprehensive explanatory model (8, 9).

However, when applying these integrated models, existing research often overlooks the contextual ecological validity of the constructs. In the specific, high-risk, and high-psychological-barrier context of sustaining exercise after a sports injury, there is substantial measurement overlap and theoretical redundancy between perceived behavioral control and self-efficacy from the classic models (10, 11). Consequently, grounded in the context of exercise rehabilitation, this study integrates and refines these two concepts into “control beliefs regarding exercise rehabilitation”. Breaking away from the traditional assumption of parallel variable relationships, we construct a chained mediation model (12). This study aims to uncover how post-injury health beliefs influence continuous exercise intentions through the chained transmission of attitude → control beliefs, thereby expanding the application boundaries of the HBM-TPB integrated model and providing evidence-based support for universities to formulate precise psychological interventions and sports rehabilitation strategies. Specifically, this study makes three contributions: (1) we challenge the traditional parallel-mediation assumption by proposing and testing a chained mediation model, clarifying the distal-to-proximal transmission of health beliefs; (2) we introduce a context-specific construct—"control beliefs regarding exercise rehabilitation”—to resolve the theoretical redundancy between perceived behavioral control and self-efficacy in the injury context; and (3) by uncovering the functional differentiation of risk perceptions (i.e., the dual-route mechanism), we provide empirically grounded targets for psychological interventions in university sports rehabilitation settings. The remainder of this paper is structured as follows: Section 2 presents the theoretical framework and hypotheses; Section 3 describes the methods; Section 4 reports the results; Section 5 discusses the findings; and Section 6 concludes with implications and limitations.

## 2 Theoretical analysis and research hypotheses

To systematically investigate this process, we first elaborate on the theoretical foundations underlying the integration of the two models.

### 2.1 Theoretical analysis

Continuous exercise intention after a sports injury is not merely a simple repetition of behavior; rather, it is a complex health behavior decision-making process involving physiological functional reconstruction and psychological adaptation following trauma. This process involves not only the proactive acquisition of rehabilitation resources but also an internal struggle to overcome reinjury fear and rebuild bodily trust.

The classic theories explaining individuals’ adoption of health-promoting behaviors primarily include the TPB and the HBM. The TPB is an explanatory framework that focuses on the behaviors individuals adopt after careful deliberation. This theory posits that behavioral intention is the most direct determinant of behavior, and intention itself is jointly driven by three core variables: the individual’s positive or negative evaluation of performing the behavior (attitude), the perceived social support and pressure from significant others (subjective norm), and the anticipated ease or difficulty of performing the behavior (perceived behavioral control) (13). Extensive meta-analyses have shown that the TPB has strong explanatory power in predicting various health behaviors, typically accounting for approximately 40 to 50% of the variance in behavioral intention (14).

While the TPB primarily focuses on volitional drive after motivation is formed, it often overlooks the motivational triggers derived from an individual’s perception of health threats and risk assessments. The HBM effectively fills this gap. As a representative theory explaining the antecedents of health behavior motivation, the HBM posits that an individual’s behavioral decisions depend on their risk perception of health threats—including the assessment of the likelihood of a health problem occurring (perceived susceptibility) and its potential consequences (perceived severity)—as well as the utility assessment of behavioral outcomes, namely the benefits of taking action (perceived health benefits) and the resistance faced (perceived barriers) (15). In the field of health behavior prevention and intervention, the HBM and TPB are recognized as two of the most explanatory and widely used theoretical frameworks (16).

A single theory struggles to fully capture the transitional process of injured students moving from the shadow of trauma to volitional action. The TPB neglects the early risk arousal associated with the threat of injury (17), while relying solely on the static constructs of the HBM fails to delineate the dynamic transmission pathways from motivation to volition (4). Integrating the distal risk assessment of the HBM with the proximal socio-cognitive pathways of the TPB can significantly enhance the predictive validity of the model for exercise and health behaviors (18); this constitutes the core logic for integrating the two in this study. Under this comprehensive framework, health beliefs in the HBM are treated as distal antecedent variables that ultimately drive continuous exercise intention through the TPB framework by shaping the individual’s attitude and subjective norm (12).

During the process of model integration, one must directly address the boundary challenges posed by the sports injury rehabilitation context to classic concepts. For a long time, there has been an academic debate regarding whether a fundamental difference exists between self-efficacy (SE) in the HBM and perceived behavioral control (PBC) in the TPB (10). Theoretically, PBC encompasses external resources and internal capabilities, whereas SE strictly focuses on internal capability beliefs (19). However, recent meta-analytical evidence indicates that in sports contexts, the correlation between the two is as high as *r* ≈ 0.60–0.70, facing significant discriminant validity overlap (11). In the specific context of this study, modern university students face relatively minor barriers to accessing external sports rehabilitation resources; the true barriers hindering behavioral execution are almost entirely internalized as psychological struggles (20). Therefore, adhering to the principle of theoretical parsimony in integration, this study does not mechanically introduce SE and PBC in parallel. Instead, it contextualizes and merges the two into a more ecologically valid core construct: “control beliefs regarding exercise rehabilitation”. This construct specifically refers to an individual’s subjective sense of mastery, during the post-injury phase, in being convinced of their ability to overcome psychological fear and physiological discomfort to persistently and safely execute rehabilitation exercises. This reconstruction not only eliminates collinear redundancy between variables but also more precisely captures the core driving force in the transition from risk perception to intention among injured students.

This decision to merge the two constructs is grounded not only in their statistical overlap—meta-analytic evidence has shown correlations between self-efficacy and perceived behavioral control as high as 0.60–0.70 in physical activity contexts (11)—but also, and more fundamentally, in the ecological characteristics of the sport injury rehabilitation setting. Within the target population of this study, university students’ access to external rehabilitation resources—such as professional guidance, structured exercise facilities, and standardized recovery protocols—is relatively homogeneous and entails low structural barriers. The genuine bottleneck that impedes behavioral execution is therefore not a shortage of external resources, but rather internalized psychological hurdles: fear of re-injury, anticipated pain, and collapsed execution confidence. Consequently, forcibly separating the two into “internal capability” and “external resource” components is not only operationally difficult to disentangle, but also theoretically unsupported in this specific context. This contextualized reconstruction represents a necessary calibration of the boundary conditions of classical constructs, rather than a mere conceptual substitution. By anchoring the merged construct specifically to the subjective mastery over internal barriers encountered during post-injury rehabilitation, we enhance both the ecological validity and the parsimony of the integrated HBM-TPB framework, while avoiding the discriminant validity issues that have plagued previous applications in other health behavior domains (10, 21).

In other health domains—such as dietary behavior or smoking cessation—the distinction between internal efficacy (resisting temptation) and external control (access to healthy food) carries practical meaning because both vary substantially across individuals. In post-injury rehabilitation, however, the external environment is highly standardized: professional protocols, prescribed exercise regimens, and scheduled follow-ups are largely uniform across students within the same institution. The only meaningful source of variation lies in the individual’s internal psychological response to these protocols—fear, pain catastrophizing, and confidence. Thus, the internal–external distinction collapses into a functionally unified construct centered on psychological mastery, making the merge not merely parsimonious but ecologically necessary.

### 2.2 Research hypotheses

**Figure 1:**
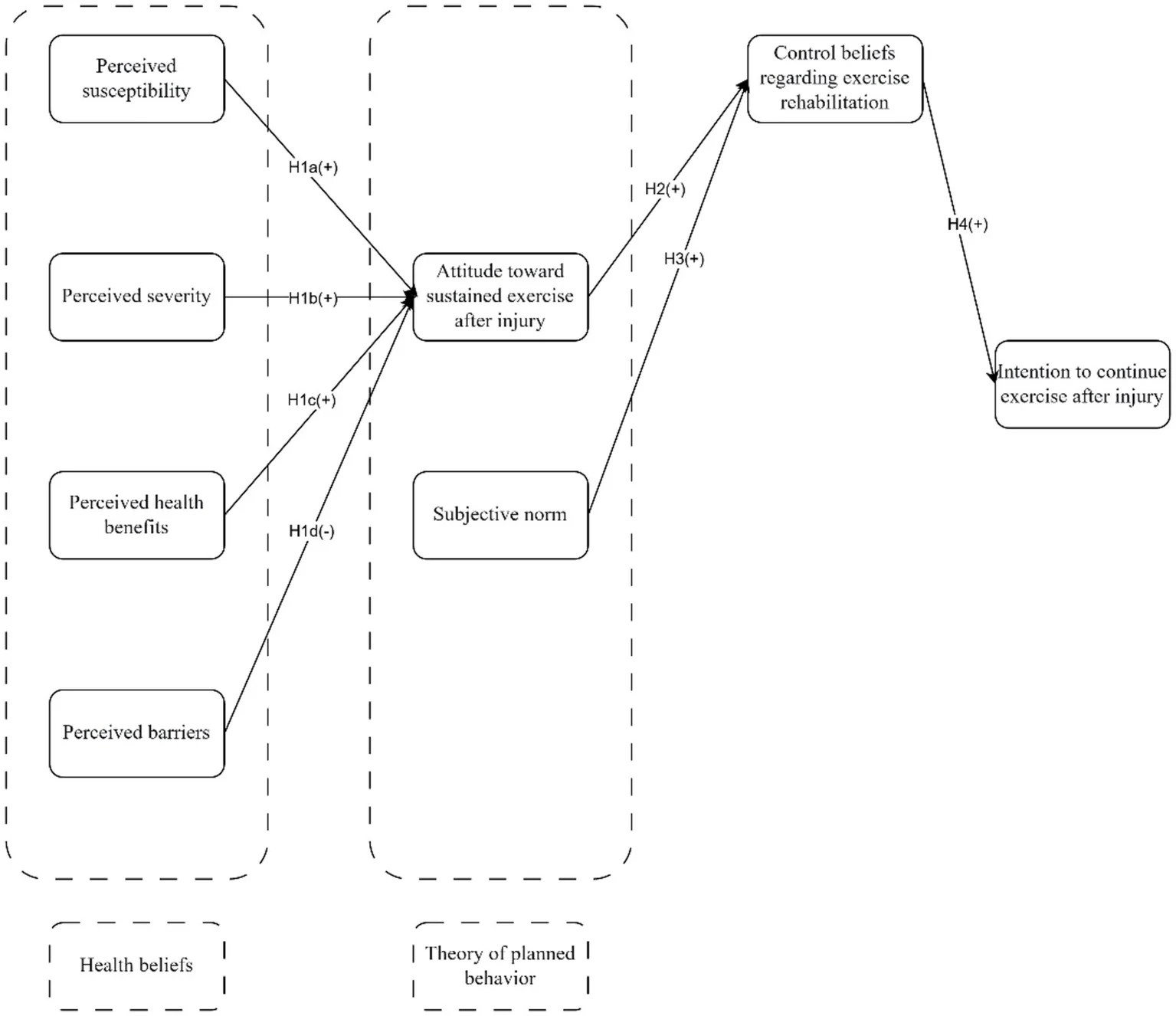
The hypothesized chain mediation model. Standardized *β* values are shown adjacent to each path. *R*^2^ values for attitude, control beliefs, and intention are 0.655, 0.499, and 0.679, respectively. Model fit: *χ*^2^/*df* = 2.799, RMSEA = 0.065, CFI = 0.907, TLI = 0.896. *p* < 0.001 for all paths shown with coefficients; the path from Perceived Barriers to Attitude was non-significant (*β* = 0.056, *p* = 0.468). *N* = 421.

Based on the integrated chained framework discussed above, this study logically deduced the hypothesized relationships among the latent variables, as illustrated in Figure 1. The HBM indicates that an individual’s risk perception of health threats (susceptibility, severity) and their utility assessment of behavioral outcomes (benefits, barriers) serve as antecedent variables explaining their motivation for health behaviors (22, 23). In the context of sports injury rehabilitation, empirical studies have shown that the aforementioned four health beliefs directly shape the injured student’s value judgment regarding the resumption of exercise, defined as attitude (24). Specifically, the higher the perceived likelihood of reinjury, the more severe the perceived consequences, or the more prominent the perceived benefits of rehabilitation exercise, the more inclined the student is to hold a positive attitude toward resuming exercise; conversely, the more perceived barriers there are, the more negative the attitude (25, 26). Accordingly, we propose:

*H1*: Health belief variables significantly influence the attitude toward sustained exercise after injury.

*H1a*: Perceived susceptibility positively influences the attitude toward sustained exercise after injury.

*H1b*: Perceived severity positively influences the attitude toward sustained exercise after injury.

*H1c*: Perceived health benefits positively influence the attitude toward sustained exercise after injury.

*H1d*: Perceived barriers negatively influence the attitude toward sustained exercise after injury.

The Theory of Planned Behavior posits that attitude and subjective norm are crucial antecedents of perceived behavioral control (13). On one hand, an individual’s positive evaluation of the behavior itself (attitude) enhances their confidence in performing it (19); on the other hand, supportive normative pressure from significant others (subjective norm) can indirectly elevate an individual’s belief in their own capabilities by providing a sense of psychological safety (27). In the high-risk context of sustaining exercise post-injury, this logic remains applicable: a positive intrinsic attitude and external social encouragement jointly constitute the core sources of “control beliefs regarding exercise rehabilitation”. Accordingly, we propose:

*H2*: Attitude toward sustained exercise after injury positively influences control beliefs regarding exercise rehabilitation.

*H3*: Subjective norm positively influences control beliefs regarding exercise rehabilitation.

Within the TPB framework, perceived behavioral control is the strongest and most direct predictor of behavioral intention, especially when the behavior itself is challenging or involves risk (13, 28). For university students who have experienced a sports injury, the ability to overcome reinjury fear and physiological discomfort, and the conviction in their capacity to safely resume exercise (i.e., control beliefs regarding exercise rehabilitation), constitute the critical psychological gateway determining whether they form a clear exercise intention. Accordingly, we propose:

*H4*: Control beliefs regarding exercise rehabilitation positively influence the intention to continue exercise after injury.

Distal variables such as health beliefs typically require the mediation of proximal psychological variables (attitude, control beliefs) to exert a substantial impact on behavioral intention (29). When integrating the HBM and TPB, attitude and control beliefs can each act as independent mediators or be linked sequentially to form a chained transmission pathway (30). Specifically, perceived health benefits may first stimulate a positive attitude, subsequently strengthening rehabilitation control beliefs, and ultimately driving behavioral intention; meanwhile, certain risk perceptions (such as susceptibility) may bypass attitude to directly erode control beliefs (27). Based on this logic, we propose the core mediation hypotheses of this study:

*H5*: Attitude and control beliefs regarding exercise rehabilitation play a significant mediating role in the model.

*H5a*: Attitude toward sustained exercise after injury mediates the relationship between health beliefs and continuous exercise intention.

*H5b*: Control beliefs regarding exercise rehabilitation mediate the relationship between health beliefs and continuous exercise intention.

*H5c*: Control beliefs regarding exercise rehabilitation mediate the relationship between subjective norm and continuous exercise intention.

*H5d*: Attitude toward sustained exercise after injury and control beliefs regarding exercise rehabilitation play a significant chained mediating role between health beliefs and continuous exercise intention.

## 3 Research methodology

### 3.1 Questionnaire design and measurement method

The design of the measurement tools in this study adhered to the principle of theoretical construct operationalization. Based on the integration of the HBM and TPB, classic scales were contextually adapted to align with the sports injury rehabilitation context of university students. All latent variables were scored using a 7-point Likert scale. The questionnaire primarily comprised the following modules: First, covariates and contextual baseline characteristics, including demographic variables and sports injury history, utilized for target group screening and controlling confounding effects. Second, antecedent motivation and risk assessment constructs based on the HBM, measuring perceived susceptibility, perceived severity, perceived health benefits, and perceived barriers to quantify the psychological cognitive imprint of the trauma experience. Constructs based on the integrated TPB for volitional transformation and intention drivers measured the post-injury sustained exercise attitude and subjective norm (detailed in Table 1). Notably, addressing the theoretical overlap between perceived behavioral control and self-efficacy in this context, this study developed the “Control Beliefs Regarding Exercise Rehabilitation Scale” based on integration logic. This scale focuses on the individual’s internal sense of mastery, ensuring they can correctly and consistently execute rehabilitation exercises when facing internal psychological barriers such as localized pain and fear of reinjury, thereby achieving a contextualized measurement of both the essence of HBM’s self-efficacy and the perceived control dimension of the TPB.

**Table 1:** Constructs and measurement items.

Theory	Variable	Measurement Items
Health Belief Model	Perceived susceptibility	1. I believe I am more susceptible to re-injury during exercise than others.
		2. I worry that if I am not careful, the same injury will occur again.
		3. I believe my physical condition makes me prone to re-injury.
	Perceived severity	1. If I am injured again, it will severely affect my sports career/hobbies.
		2. I believe that a re-injury would cause trouble for my studies/work.
		3. I worry that a re-injury will permanently prevent me from returning to my previous sports performance level.
		4. I am afraid of being injured again while exercising, and this worry often comes to mind.
	Perceived health benefits	1. I believe that gradually resuming exercise after an injury can help me recover faster.
		2. I believe that continuous exercise can enhance my physical resistance and prevent re-injury.
		3. I believe that appropriate exercise can improve my psychological state following an injury.
		4. I believe that persistent exercise is essential for maintaining overall health.
	Perceived barriers	1. I am worried about re-injury during exercise, which makes me hesitant to start.
		2. I find it difficult to maintain the strict self-discipline required to execute a post-injury recovery exercise plan.
		3. I worry that even if I exercise diligently, the rehabilitation effects will not be significant.
		4. I feel a lack of a supportive atmosphere around me for resuming exercise after an injury.
Theory of Planned Behavior	Attitude toward sustained exercise after injury	1. I believe that gradually resuming exercise after an injury is a good idea.
		2. I think it is wise to continue exercising under professional guidance after an injury.
		3. I hold a positive attitude toward recovering and sustaining exercise post-injury.
		4. I believe that continuing to exercise after an injury is worth the investment of time and energy.
	Subjective norm	1. My coach/physical education teacher supports my resumption of exercise after an injury.
		2. My teammates/classmates believe that I should gradually resume exercise following an injury.
		3. My family supports my engagement in exercise under rehabilitation guidance after an injury.
Mediating variable	Control beliefs regarding exercise rehabilitation	1. I am confident that I can stick to and complete my post-injury recovery exercise plan.
		2. I have the confidence to persist in post-injury recovery exercise over the long term.
		3. I am confident in my ability to correctly perform post-injury recovery exercises.
		4. I have the confidence to overcome difficulties encountered during post-injury exercise.
		5. The time and energy costs of continuous post-injury exercise are within my acceptable limits.
Outcome variable	Intention to continue exercise after injury	1. Within the next 3 months, I intend to gradually recover and sustain my exercise routine.
		2. I plan to participate in post-injury recovery exercises regularly in the future.
		3. I intend to make exercise an important part of my post-injury rehabilitation.
		4. I intend to persist in long-term exercise to prevent re-injury.

To ensure the content validity of the questionnaire, the initial draft was reviewed by a panel of three experts in health psychology and behavioral science. The experts independently evaluated each item based on its relevance, clarity, and representativeness to the corresponding construct, and minor adjustments were made to address potentially overlapping items. Subsequently, a pilot test was conducted with 50 eligible university students to assess the clarity and readability of the questionnaire within the target population. The preliminary reliability assessment indicated that the initial internal consistency of each construct was satisfactory, with no items requiring deletion. Based on the open-ended feedback from the participants, the research team only optimized the wording of certain items to improve contextual expression and enhance respondents’ comprehension accuracy. The content coverage and construct validity of the final version of the scale were fully confirmed in the subsequent confirmatory factor analysis (CFA) and reliability and validity tests using the formal large sample (*N* = 421) (see Section 4.3).

### 3.2 Data collection and survey participants

This study adopted a cross-sectional survey design and was conducted at Nanjing Normal University from April 10 to April 30, 2026. A combined strategy of convenience and purposive sampling was employed, recruiting respondents through both online (Wenjuanxing platform, campus social groups) and offline (sports venues, rehabilitation studios) channels. Inclusion criteria were: (1) full-time enrolled university students; (2) having experienced at least one sports injury leading to exercise interruption within the past 12 months; and (3) possessing independent reading and comprehension capabilities. Exclusion criteria included individuals with medical contraindications to exercise and those who failed attention-check questions.

During data collection, social desirability bias was mitigated through promises of anonymity and instructions stating there were no right or wrong answers. In the data cleaning phase, questionnaires with excessively short or long completion times, consecutive identical responses, failed attention checks, or logical contradictions were excluded. Out of 512 initially collected questionnaires, 421 were deemed valid, yielding an effective rate of 82.2%. The sample comprised 129 males (30.6%) and 292 females (69.4%), predominantly undergraduate (53.2%) and master’s students (46.3%), with non-sports majors accounting for 83.1%. The most common type of recent injury was muscle strain/contusion (49.4%), as detailed in Table 2.

**Table 2:** Demographic characteristics of the participants.

Variable	Category	Frequency (n)	Percentage (%)
Gender	Male	129.00	30.60%
	Female	292.00	69.40%
Educational level	Undergraduate	224.00	53.20%
	Master’s student	195.00	46.30%
	Doctoral student	2.00	0.50%
Major	Sports-related major	71.00	16.90%
	Other majors	350.00	83.10%
Years of exercise	< 1 year	155.00	36.80%
	1–3 years	107.00	25.40%
	3–5 years	34.00	8.10%
	> 5 years	125.00	29.70%
Primary type of recent sports injury	Muscle strain/contusion	208.00	49.40%
	Ligament injury	64.00	15.20%
	Fracture	8.00	1.90%
	Joint injury	29.00	6.90%
	Chronic strain	45.00	10.70%
	Other soft tissue injuries	67.00	15.90%

### 3.3 Data processing

Data analysis was conducted using SPSS 26.0 and AMOS 26.0. Harman’s single-factor test was used to assess common method bias; Confirmatory Factor Analysis (CFA) was employed to test the reliability and validity of the measurement model; Structural Equation Modeling (SEM) was estimated using the maximum likelihood method to test direct path hypotheses; and the PROCESS macro (Models 4 and 6) along with a bias-corrected non-parametric percentile Bootstrap method were utilized to test the mediation effects (31). In the present structural equation modeling (SEM) analysis, we did not include demographic variables (gender, educational level, major, and injury type) as covariates. The decision was based on the following considerations: As shown in the correlation matrix (Table 3), the absolute values of the correlations between these demographic variables and the core latent constructs—attitude toward sustained exercise, control beliefs regarding exercise rehabilitation, and intention to continue exercise—were all below 0.20, and the vast majority did not reach statistical significance (*p* > 0.05). Forcing a large number of low-correlated covariates into the SEM would not only increase model complexity by consuming degrees of freedom, but could also artificially inflate fit indices or produce unstable parameter estimates (32). Therefore, in line with the principle of parsimony and while preserving the integrity of the theoretical constructs, we retained the most concise structural model without covariate adjustment. The descriptive statistics of these demographic variables are reported in Table 2, and their potential moderating effects are acknowledged as a limitation for future research (see Section 5.5).

**Table 3:** Means, standard deviations, and Pearson correlation coefficient matrix.

Variable	M	SD	1	2	3	4	5	6	7	8
1. Perceived susceptibility	4.02	1.34	1							
2. Perceived severity	4.33	1.36	0.565**	1						
3. Perceived health benefits	5.38	1	0.023	0.185**	1					
4. Perceived barriers	3.75	1.31	0.479**	0.492**	−0.118*	1				
5. Attitude toward sustained exercise	5.5	1.03	0.087	0.109*	0.588**	−0.065	1			
6. Subjective norm	5.12	1.19	0.039	0.076	0.490**	−0.109*	0.654**	1		
7. Control beliefs regarding exercise rehabilitation	5.08	1.2	−0.107*	−0.008	0.465**	−0.291**	0.584**	0.571**	1	
8. Intention to continue exercise	5.2	1.16	−0.044	0.064	0.537**	−0.196**	0.636**	0.570**	0.750**	1

**p < 0.01, *p < 0.05(two-tailed); *N* = 421.

## 4 Results

### 4.1 Common method bias testing

Given that all data in this study were derived from self-reports by the respondents, there was a potential risk of common method bias (33). To evaluate the severity of this bias, Harman’s single-factor test was employed to statistically assess the data. All measurement items of the core variables in the questionnaire were included in an unrotated exploratory factor analysis. The results revealed 6 factors with eigenvalues greater than 1, where the first principal component explained 32.998% of the variance, strictly falling below the critical threshold of 50%. This result indicates that there is no single dominant factor in the study data, meaning the issue of common method bias is within an acceptable range and will not exert a substantive impact on subsequent hypothesis testing.

It should be noted that Harman‘s single-factor test, as a preliminary screening method, has limited diagnostic power. However, the core conclusions of this study are primarily based on structural relationships among latent variables rather than comparisons of individual item means, and the subsequent covariate sensitivity analysis (Section 4.6) further supports the robustness of the model. Therefore, the threat of common method bias to the study’s conclusions remains within a manageable scope.

### 4.2 Descriptive statistics and correlation analysis

The means, standard deviations, and Pearson correlation coefficients of each variable are presented in Table 3. The surveyed university students scored highest on sustained exercise attitude (*M* = 5.50, SD = 1.03) and perceived health benefits (*M* = 5.38, SD = 1.00), and lowest on perceived barriers (*M* = 3.75), overall exhibiting characteristics of high value identification and moderate risk perception. Continuous exercise intention was significantly and positively correlated with perceived health benefits (*r* = 0.537), attitude (*r* = 0.636), subjective norm (*r* = 0.570), and control beliefs (*r* = 0.750) (*p* < 0.01), and significantly negatively correlated with perceived barriers (*r* = −0.196, *p* < 0.01). Notably, the zero-order correlations of perceived susceptibility (*r* = −0.044) and perceived severity (*r* = 0.064) with continuous exercise intention were not significant. All variable correlation coefficients were below 0.80, indicating the absence of severe multicollinearity issues.

### 4.3 Measurement model testing: reliability and validity

Prior to the SEM path analysis, this study first systematically assessed the goodness-of-fit, reliability, and validity of the measurement model through confirmatory factor analysis (CFA), as shown in Table 4. The model fit indices showed: *χ^2^/df* = 2.432 (< 3.0), RMSEA = 0.058 (< 0.08), CFI = 0.928, TLI = 0.917, and IFI = 0.928 (all > 0.90). All core fit indices surpassed accepted academic standards, indicating an excellent fit between the constructed measurement model and the empirical data.

**Table 4:** Reliability and convergent validity of the measurement model.

Latent variable	*λ*	Cronbach’s *α*	CR	AVE
Perceived susceptibility	0.561–0.893	0.78	0.796	0.574
Perceived severity	0.683–0.745	0.795	0.797	0.496
Perceived health benefits	0.632–0.786	0.801	0.815	0.526
Perceived barriers	0.669–0.819	0.804	0.806	0.512
Attitude toward sustained exercise after injury	0.707–0.832	0.85	0.852	0.591
Subjective norm	0.774–0.884	0.849	0.856	0.665
Control beliefs regarding exercise rehabilitation	0.817–0.924	0.935	0.936	0.745
Intention to continue exercise after injury	0.782–0.879	0.904	0.905	0.705

All item loadings were highly significant at *p* < 0.001.

Regarding reliability and convergent validity, comprehensive evaluations were conducted using factor loadings (*λ*), Cronbach’s α coefficients, Composite Reliability (CR), and Average Variance Extracted (AVE), with results in Table 4. The Cronbach’s α coefficients for all latent variables ranged from 0.780 to 0.935, and CR values ranged from 0.796 to 0.936, all exceeding the recommended threshold of 0.70, demonstrating excellent internal consistency. Concurrently, the standardized factor loadings for all observed items ranged from 0.561 to 0.924 (all highly significant at *p* < 0.001). Notably, the AVE value for the perceived severity dimension was 0.496, slightly below the ideal threshold of 0.50; however, because its CR value reached 0.797 (significantly above 0.60), the convergent validity of this construct remains acceptable according to the recommendations of Fornell and Larcker (34). The AVE values for all other latent variables exceeded 0.50. In summary, the scale demonstrated excellent reliability and convergent validity.

Furthermore, the Fornell-Larcker criterion was applied to test the discriminant validity of the model. As shown in Table 5, the bolded values on the diagonal represent the square root of the AVE for each latent variable (ranging from 0.704 to 0.863), which are all greater than the correlation coefficients between that latent variable and other latent variables in the same row and column. This result provides robust evidence that the 8 latent variables involved in this study possess excellent statistical discriminant validity; the boundaries between constructs are clear, and there is no severe collinearity or measurement overlap.

**Table 5:** Discriminant validity of the measurement model.

Latent variables	1	2	3	4	5	6	7	8
Perceived susceptibility	**0.758**							
Perceived severity	0.63	**0.704**						
Perceived health benefits	−0.059	0.211	**0.725**					
Perceived barriers	0.568	0.599	−0.181	**0.716**				
Attitude toward sustained exercise after injury	0.062	0.133	0.693	−0.114	**0.769**			
Subjective norm	0.014	0.093	0.565	−0.144	0.755	**0.815**		
Control beliefs regarding exercise rehabilitation	−0.146	−0.001	0.527	−0.342	0.666	0.621	**0.863**	
Intention to continue exercise after injury	−0.091	0.083	0.606	−0.229	0.727	0.643	0.813	**0.840**

The bolded values on the diagonal represent the square roots of the AVE for each latent variable, while the remaining values represent the correlation coefficients between the variables.

### 4.4 Structural model and direct hypothesis testing

**Figure 2:**
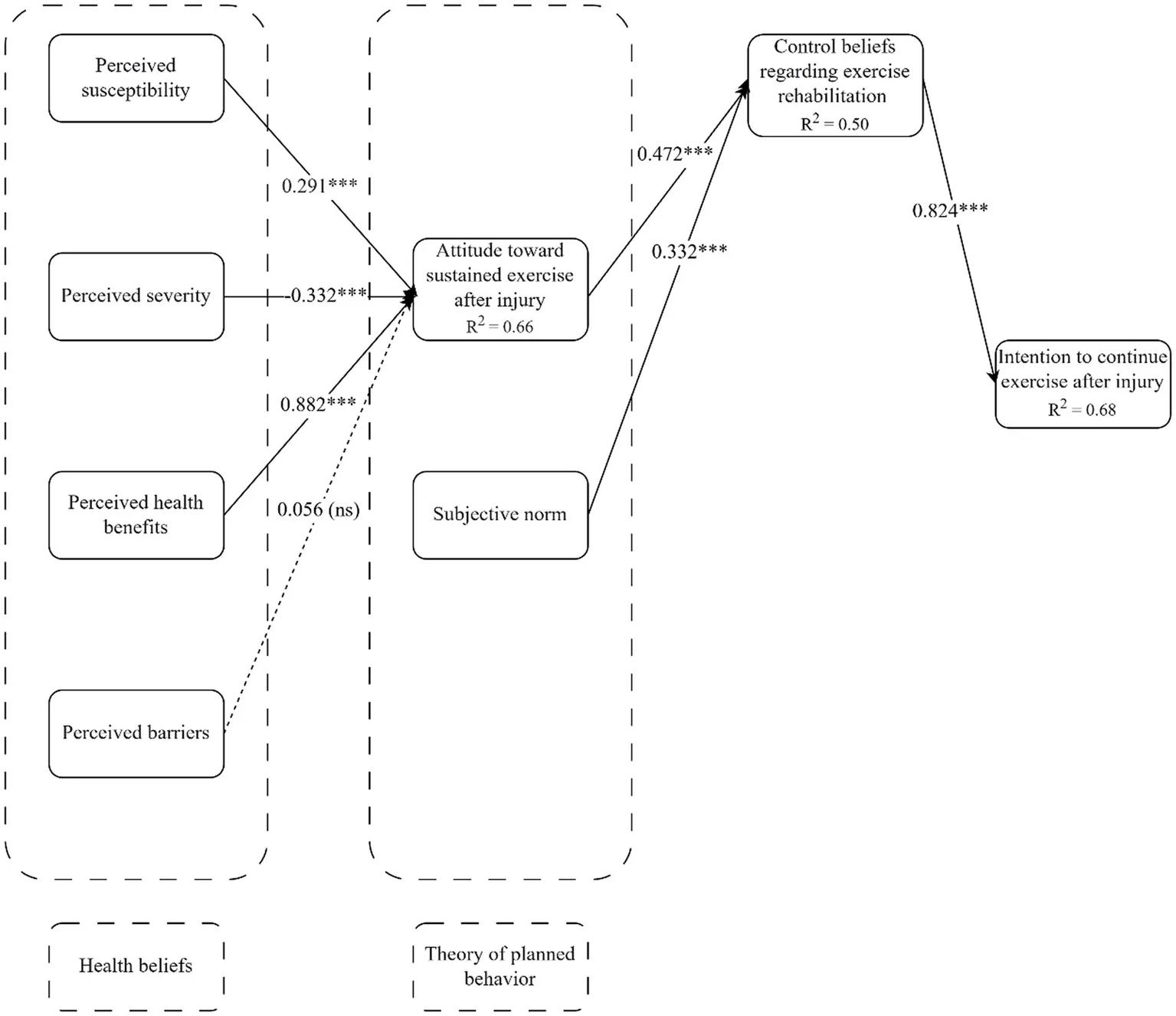
Results of the structural equation model with standardized path coefficients. ***p < 0.001.

After confirming the robust reliability and validity of the measurement model, a structural equation model was constructed to test the hypothesized path relationships among the latent variables within the theoretical framework, as depicted in Figure 2. The maximum likelihood method was employed in AMOS 26.0 to estimate the structural model. The goodness-of-fit test results for the model showed: *χ^2^/df* = 2.799, RMSEA = 0.065, CFI = 0.907, TLI = 0.896, and IFI = 0.907. These indices collectively indicated acceptable model fit, with most core fit indices meeting or approaching conventional benchmarks.

The TLI value was 0.896, marginally below the conventional 0.90 benchmark. However, given that CFI (0.907) and RMSEA (0.065) both fell within acceptable ranges, and considering the complexity of the chained mediation model (eight latent variables with multiple pathways), the overall model fit is deemed acceptable (35). We did not rely on modification indices for post-hoc adjustments to avoid capitalizing on chance, thereby preserving the theoretical integrity of the model.

Based on this structural model, the direct path effects among the variables were tested, with detailed hypothesis testing results presented in Table 6.

**Table 6:** Results of direct path analysis and hypothesis testing in the structural equation model.

Hypothesis	Path relationship	Standardized path coefficient (*β*) **↑**	Standard error (S. E.) **↓**	Critical ratio (C. R.) **↑**	*p*-value**↓**	Test result
H1a	Perceived Susceptibility → Attitude Toward Continued Exercise After Injury	0.291	0.043	3.911	***	Supported
H1b	Perceived Severity → Attitude Toward Continued Exercise After Injury	−0.332	0.066	−3.442	***	Not Supported (Significantly Negative)
H1c	Perceived Health Benefits → Attitude Toward Continued Exercise After Injury	0.882	0.105	10.031	***	Supported
H1d	Perceived Barriers → Attitude Toward Continued Exercise After Injury	0.056	0.057	0.726	0.468	Not Supported (Not Significant)
H2	Attitude Toward Continued Exercise → Control beliefs regarding exercise rehabilitation	0.472	0.083	8.105	***	Supported
H3	Subjective Norm → Control beliefs regarding exercise rehabilitation	0.322	0.064	5.916	***	Supported
H4	Control beliefs regarding exercise rehabilitation → Intention to Continue Exercise After Injury	0.824	0.042	18.364	***	Supported

*** indicates *p* < 0.001; in structural equation modeling, the Critical Ratio (C. R.) is equivalent to the *t*-value.

In the first phase of paths from health beliefs to attitude, perceived susceptibility had a significant positive impact on the post-injury sustained exercise attitude (*β* = 0.291, *p* < 0.001), and perceived health benefits significantly and positively predicted attitude (*β* = 0.882, *p* < 0.001); thus, hypotheses H1a and H1c were supported. The influence of perceived severity on attitude exhibited a significant negative effect (*β* = −0.332, *p* < 0.001), contrary to the positive expectation of hypothesis H1b; hence, H1b was not supported. The path of perceived barriers influencing attitude did not reach statistical significance (*β* = 0.056, *p* = 0.468 > 0.05); therefore, H1d was not supported. Overall, hypothesis H1 received partial support.

In the second phase of paths from attitude and subjective norm to control beliefs, attitude toward sustained exercise after injury significantly and positively predicted control beliefs regarding exercise rehabilitation (*β* = 0.472, *p* < 0.001); subjective norm significantly and positively influenced control beliefs regarding exercise rehabilitation (*β* = 0.322, *p* < 0.001). Hypotheses H2 and H3 were both supported.

In the third phase path from control beliefs to behavioral intention, control beliefs regarding exercise rehabilitation significantly and positively predicted the intention to continue exercise after injury (*β* = 0.824, *p* < 0.001). Hypothesis H4 was supported.

Furthermore, the explanatory power analysis for the endogenous variables revealed that the squared multiple correlations (*R^2^*) for sustained exercise attitude, control beliefs regarding exercise rehabilitation, and continuous exercise intention were 0.655, 0.499, and 0.679, respectively.

### 4.5 Multiple and chained mediation effects testing

Using the SPSS PROCESS macro, with perceived susceptibility, perceived severity, perceived health benefits, and perceived barriers as independent variables respectively, the multiple and chained mediation effects of post-injury sustained exercise attitude and exercise rehabilitation control beliefs were tested utilizing the bias-corrected non-parametric percentile Bootstrap method (detailed in Table 7). For clarity in interpreting the mediation results, the three indirect paths are designated as follows: Ind1 represents the indirect effect of the predictor on intention transmitted exclusively through attitude; Ind2 represents the indirect effect transmitted exclusively through control beliefs; and Ind3 represents the chained indirect effect transmitted sequentially through attitude and then control beliefs.

**Table 7:** Results of multiple and chained mediation analyses based on bootstrap sampling.

Predictor variable	Path name	Mediation path	Effect Value	Standard Error (S. E.)	BootLLCI	BootULCI	Result
Perceived Health Benefits (X3)	Independent Mediation 1 (Ind1)	X3 → Attitude → Intention	0.1568	0.0438	0.0742	0.2470	Significant
	Independent Mediation 2 (Ind2)	X3 → Control beliefs → Intention	0.1182	0.0387	0.049	0.2001	Significant
	Chained Mediation (Ind3)	X3 → Attitude →Control beliefs → Intention	0.1778	0.0351	0.1135	0.2500	Significant
Perceived Susceptibility (X1)	Independent Mediation 1 (Ind1)	X1 → Attitude → Intention	0.0227	0.0138	−0.0037	0.0507	Not Significant
	Independent Mediation 2 (Ind2)	X1 → Control beliefs → Intention	−0.0789	0.0238	−0.1287	−0.0344	Significant
	Chained Mediation (Ind3)	X1 → Attitude →Control beliefs → Intention	0.0257	0.0166	−0.004	0.0614	Not Significant
Perceived Barriers (X4)	Independent Mediation 1 (Ind1)	X4 → Attitude → Intention	−0.0175	0.015	−0.048	0.0120	Not Significant
	Independent Mediation 2 (Ind2)	X4 → Control beliefs → Intention	−0.1294	0.0252	−0.1803	−0.0821	Significant
	Chained Mediation (Ind3)	X4 → Attitude →Control beliefs → Intention	−0.0188	0.0162	−0.0515	0.0124	Not Significant
Perceived Severity (X2)	Independent Mediation 1 (Ind1)	X2 → Attitude → Intention	0.0275	0.0153	−0.0005	0.0597	Not Significant
	Independent Mediation 2 (Ind2)	X2 → Control beliefs → Intention	−0.0362	0.0235	−0.0836	0.0082	Not Significant
	Chained Mediation (Ind3)	X2 → Attitude →Control beliefs → Intention	0.0321	0.0184	−0.0007	0.0710	Not Significant
Subjective Norm (SN)	Simple Mediation (Ind)	SN → Control beliefs → Intention	0.3505	0.0529	0.2504	0.4597	Significant

A total of 5,000 bootstrap resamples were generated for the mediation analyses. BootLLCI and BootULCI refer to the lower and upper bounds of the 95% bias-corrected confidence interval, respectively. Statistical significance is established when the 95% confidence interval excludes zero.

First, when perceived health benefits were the predictor variable, all three mediation paths were significant. The effect size for Ind1 (X3 → Attitude → Intention) was 0.1568 (95% CI [0.0742, 0.2470]); the effect size for Ind2 (X3 → Control Beliefs → Intention) was 0.1182 (95% CI [0.0490, 0.2001]); and the chained mediation effect size for Ind3 (X3 → Attitude → Control Beliefs → Intention) was 0.1778 (95% CI [0.1135, 0.2500]), representing the largest effect among all indirect paths.

Second, when perceived susceptibility or perceived barriers served as the predictor variable, the Bootstrap confidence intervals for the chained mediation and the independent attitude mediation paths both included zero, indicating non-significant effects. The independent mediation of control beliefs was significant for both, with effect sizes of −0.0789 [95% CI (−0.1287, −0.0344)] and −0.1294 [95% CI (−0.1803, −0.0821)], respectively.

Third, when testing perceived severity independently, the 95% confidence intervals for all three mediation paths included zero, failing to reach significance. The simple mediation effect of subjective norm was significant, with an effect size of 0.3505 [95% CI (0.2504, 0.4597)].

In summary, all three mediation paths for perceived health benefits, alongside the indirect effect of subjective norm, were significant; for perceived susceptibility and perceived barriers, only the independent mediation path of control beliefs was significant; for perceived severity, all mediation paths were non-significant.

### 4.6 Covariate sensitivity analysis

To examine potential confounding effects of demographic variables and injury types on the model’s conclusions, two supplementary models were constructed based on the baseline structural model.

Model 1 (M1) entered gender, major, and years of exercise as covariates pointing to all endogenous variables. Model fit results showed: *χ^2^/df* = 2.569, CFI = 0.908, RMSEA = 0.061, TLI = 0.893. All core paths remained significant: perceived health benefits → attitude (*β* = 0.929, *p* < 0.001), attitude → control beliefs (*β* = 0.448, *p* < 0.001), and control beliefs → intention (*β* = 0.817, *p* < 0.001). All covariate paths were non-significant (*p* > 0.05). R^2^ values were 0.689 for attitude, 0.509 for control beliefs, and 0.687 for intention.

Model 2 (M2) entered five dummy variables representing injury types (with muscle strain/contusion as the reference group) as covariates pointing to all endogenous variables. Model fit results showed: *χ^2^/df* = 2.444, CFI = 0.906, RMSEA = 0.059, TLI = 0.889. All core paths remained significant: perceived health benefits → attitude (*β* = 0.935, *p* < 0.001), attitude → control beliefs (*β* = 0.458, *p* < 0.001), and control beliefs → intention (*β* = 0.822, *p* < 0.001). All five injury-type dummy variables were non-significant predictors of attitude, control beliefs, and intention (*p* > 0.05). *R*^2^ values were 0.689 for attitude, 0.504 for control beliefs, and 0.691 for intention.

Table 8 presents a comparison of path coefficients and *R*^2^ across the three models.

**Table 8:** Sensitivity analysis: comparison of core path coefficients across three models.

Path	Original *β*	M1 *β* (+ gender/major/years)	M2 *β* (+ injury type)
Perceived health benefits → Attitude	0.882***	0.929***	0.935***
Perceived severity → Attitude	−0.332***	−0.456***	−0.470***
Perceived susceptibility → Attitude	0.291***	0.317***	0.342***
Perceived barriers → Attitude	0.056	0.175	0.148
Attitude → Control beliefs	0.472***	0.448***	0.458***
Subjective norm → Control beliefs	0.322***	0.331***	0.323***
Control beliefs → Intention	0.824***	0.817***	0.822***
*R*^2^ (Attitude) **↑**	0.655	**0.689**	**0.689**
*R*^2^ (Control beliefs) **↑**	0.499	**0.509**	0.504
*R*^2^ (Intention) **↑**	0.679	0.687	**0.691**

M1 represents the model with gender, major, and years of exercise entered as covariates; M2 represents the model with five dummy variables representing injury types (with muscle strain/contusion as the reference group) entered as covariates. ***p < 0.001. All covariate paths in both M1 and M2 were non-significant (*p* > 0.05). Bold values indicate the highest *R*^2^ value for each endogenous variable across the three models.

## 5 Discussion

### 5.1 The dual-route mechanism from cognition to intention: functional differentiation of the attitude and belief paths

In existing integrated HBM-TPB research, the dimensions of the HBM are typically treated as parallel predictor variables, operating under the assumption that their mechanistic influence on behavioral intention is homogenous and additive (36). However, the chained mediation analysis in this study reveals a more complex landscape: the transmission of various health belief dimensions toward continuous exercise intention diverges along an attitude path and a direct belief path, exhibiting distinct triggering conditions and functional properties.

Perceived health benefits exert their influence through a complete chained transmission of “attitude → control beliefs → intention” (Ind3 = 0.1778). This finding aligns with the conclusions of Li and Cho (2024), based on a sample of 5,109 students across 52 Chinese universities, suggesting that the impact of health beliefs on exercise adherence is primarily realized through the mediating channels of planned behavior and behavioral control, rather than directly affecting the behavior itself (37). A qualitative study by Marmura et al. (38) exploring the reconstruction process of young athletes post-ACL injury also revealed that individuals capable of positively reframing their injury experience reported more favorable perceptions of rehabilitation outcomes and quality of life, implying that cognitive reframing is a crucial psychological prerequisite for rebuilding active rehabilitation confidence (38). Building upon this, our study further uncovers that the translation of benefit cognition into intention is not instantaneous; rather, it is a step-wise process of “cognitive evaluation → emotional identification → efficacy conviction”. A disruption at any link results in a failure to mobilize behavior. This explains why mere rehabilitation education often yields minimal results if not simultaneously accompanied by efficacy cultivation—because the information only reaches the first tier of cognitive evaluation and fails to transmit downstream to efficacy and intention.

In stark contrast to the attitude path, the mediation effects of perceived susceptibility and perceived barriers entirely bypass the attitude component. This finding challenges the causal logic embedded in classic HBM assumptions that “risk perception alters behavioral evaluation”. Taflinger and Sattler (39), in a large-sample (*N* = 4,802) scenario experiment, noted that the impact of perceived susceptibility on preventive behavior is not consistently stable, and its effect size and direction are moderated by cognitive factors such as perceived benefits (39). This suggests a complex interaction between risk cognition and efficacy. The data from our study provide a more nuanced mechanistic explanation for this effect: the attenuation of intention by perceived susceptibility and perceived barriers is not achieved by altering the individual’s value judgment regarding the merits of rehabilitation exercise, but rather by directly eroding their “I can do it” execution beliefs. Specifically, both direct belief paths (perceived susceptibility → control beliefs → intention, Ind2 = −0.0789; perceived barriers → control beliefs → intention, Ind2 = −0.1294) reached significance, while all mediation paths originating with attitude were non-significant. This phenomenon of “zero attitude involvement” points to a specific psychological erosion mechanism: the catastrophic worry of reinjury and the tangible resistance of rehabilitation barriers dismantle execution confidence before the individual even begins to waver in their value evaluation of exercise. In other words, at a rational level, the injured student still acknowledges that rehabilitation exercise is beneficial and valuable; however, anxious expectations and execution resistance plunge them into a state of cognitive dissonance—“knowing what to do but feeling powerless to do it”—ultimately dissolving the internal conflict by abandoning the intention. This psychological state of “evaluation-confidence separation” provides a deep psychological explanation for the intention-behavior gap commonly observed in sports injury rehabilitation (40), and it demonstrates that the mechanisms forming behavioral intentions in high-risk contexts differ fundamentally from those of general health behaviors.

To clarify, we term this state “evaluation-confidence dissociation” —a psychological disconnection in which injured students, while rationally recognizing the benefits and value of rehabilitation exercise, simultaneously experience a profound deficit in their belief that they can actually execute the behavior. This misalignment between outcome valuation and agency conviction prevents the translation of positive attitudes into actionable intentions.

Why do perceived barriers bypass attitude entirely? We propose that barriers and attitudes operate at fundamentally different levels of psychological processing. Attitude represents a deliberative, evaluative judgment about the value of a behavior—a cognitive operation that engages reflective reasoning. In contrast, barrier perception is inherently experiential and proximal—it is grounded in immediate, concrete obstacles (e.g., “I feel pain when I move,” “I have no time today”). This proximity means that barriers trigger implementation-focused cognitive processes rather than value-focused ones. An injured student does not need to re-evaluate whether exercise is worthwhile to recognize that pain or scheduling conflicts make it difficult to execute; the barrier directly activates problem-solving or avoidance heuristics. Consequently, barrier perception erodes control beliefs by amplifying perceived difficulty, while leaving the individual’s rational endorsement of exercise benefits intact. This dissociation has practical implications: interventions targeting barriers should focus on coping planning (e.g., time-management strategies, pain-contingent exercise modifications) rather than health education, which primarily targets attitudes.

### 5.2 The reverse effect of perceived severity: contextual activation of the fear-avoidance mechanism

Perceived severity exhibited a significant negative influence on attitude (*β* = −0.332, *p* < 0.001), which contradicts the core HBM assumption that “severity cognition linearly enhances motivation”. However, this anomalous finding is not isolated. Li and Xia also found in a study of college students’ exercise behavior that perceived severity of illness and frailty had a significant negative effect on exercise volume, entirely mediated by physical evaluation self-efficacy (41). This study replicated this reverse effect within the context of sports injury rehabilitation and further pinpointed its operative mechanism: unlike benefit cognition, severity cognition does not translate into intention via a complete chained transmission; rather, it directly suppresses the formation of a positive attitude at the front end of the model. This finding provides consistent evidence for the dual-route functional differentiation framework proposed in this study. Unlike benefit cognition, which acts as a driver via the attitude path, severity cognition targets the same value judgment of “whether resuming exercise is worthwhile”, but its effect acts in the opposite direction. Therefore, it directly impacts attitude rather than detouring through the belief path.

Witte’s Extended Parallel Process Model (EPPM) provides a classic framework for explaining this inhibitory effect: when perceived threat exceeds coping efficacy, individuals initiate a fear control process, manifesting as defensive avoidance and attitude reversal (42). In the discourse of sports injuries, the items measuring perceived severity precisely capture the composite psychological state of low coping efficacy embedded within catastrophic cognition. Sheean et al. highlighted that fear of reinjury is the leading reason for failure to return to sport (43), and Walsh et al. further confirmed in their integrative model that injury-related fear and fear-avoidance beliefs are core predictors leading patients to abandon rehabilitation plans, independent of the objective clinical assessment of injury severity (44). The innovative contribution of the present study lies in empirically demonstrating that perceived severity operates as a cognitive antecedent to kinesiophobia, rather than merely co-occurring with it. While Ambegaonkar et al. (3) systematically documented the prevalence of kinesiophobia across injury types, our findings specify why and how catastrophic injury appraisals translate into behavioral avoidance—by directly suppressing rehabilitation attitude at the front end of the decision-making chain. This extends Sheean et al.’s (43) clinical observation that fear of reinjury is the leading reason for failed return-to-sport, by pinpointing perceived severity as a modifiable cognitive target that precedes fear development. Unlike Walsh et al. (44), who focused on the integrative role of fear-avoidance in adherence, our results suggest that interventions should target threat appraisal before fear beliefs crystallize, thereby preventing the attitude suppression that impedes rehabilitation engagement. This provides an explanatory cognitive antecedent pathway for understanding the reinjury anxiety and kinesiophobia that emerge post-injury, and empirically substantiates the theoretical proposition that fear appeals are not unconditionally effective in high-risk scenarios.

Furthermore, a methodological phenomenon revealed by this study also offers theoretical insights: perceived severity was non-significant across all paths in an independent two-variable mediation model; its negative effect was only exposed after controlling for other health belief variables. This statistical suppression effect indicates that the effects of positive variables like perceived benefits obscured the true destructive power of severity cognition during isolated testing. This finding suggests that future studies examining the motivational effects of risk perception must employ multivariate models rather than zero-order correlations; otherwise, they risk drawing the misleading conclusion that risk perception is unrelated to behavioral intention.

### 5.3 The core hub status of control beliefs regarding exercise rehabilitation: theoretical implications of contextual construct reconstruction

Self-efficacy in the HBM and perceived behavioral control in the TPB have long endured theoretically blurred boundaries. Conner and Armitage noted severe overlap between the two at both conceptual and operational levels early on, and recent meta-analyses have confirmed correlations between the two as high as 0.60–0.70 in sports contexts, presenting challenges to discriminant validity (21). Based on the ecological characteristics of sports injury rehabilitation, this study integrated the two into a unified construct: “control beliefs regarding exercise rehabilitation”, and proved the feasibility of this contextualized fusion through excellent reliability and validity metrics.

Theoretically, the reconstruction of this construct achieves a threefold contribution. First, regarding the construct itself, it resolves the measurement redundancy caused by the forced separation of self-efficacy and perceived behavioral control within this specific context. Because modern university students face relatively low barriers to standardized external rehabilitation resources, the core resistance hindering behavioral execution is almost entirely internalized into psychological hurdles such as fear of reinjury and perceived execution difficulties (6). By focusing item design on the sense of mastery over these internal barriers, the construct effectively anchors the most critical psychological gateway in the injured student’s transition from cognitive assessment to volition. Second, concerning functional model positioning, the mediating role of control beliefs shifts from “parallel mediation” in traditional models to a “core hub” in this model—positive cognitive resources (benefits, subjective norm) must translate through it, and negative cognitive resources (barriers, susceptibility) primarily target it for erosion. This aligns closely with Bandura’s theoretical proposition that self-efficacy exerts a central regulatory function in health behaviors (19); however, this study grounds it from a generalized theoretical concept into an empirically testable core hub node within the model structure, achieving a leap from theoretical assertion to empirical validation. Third, regarding practical intervention strategies, this positioning establishes confidence rebuilding as an irreplaceable core in sports rehabilitation psychological interventions, providing clear prioritization criteria for selecting targets in subsequent intervention protocols.

### 5.4 The social internalization effect of subjective norms: from external pressure to internal mastery

Subjective norm exerted a significant indirect effect on behavioral intention through control beliefs (Ind = 0.3505), forming an interesting contrast with Armitage and Conner’s classic conclusion that subjective norm is the “weakest predictor” within the TPB model (28). Our data suggests that the impact of subjective norm on intention is not as weak as traditionally believed; instead, its effect is fully mediated by control beliefs. Social support is first absorbed and reorganized by the individual into an internal sense of mastery (“I am not facing this alone”), which is subsequently transformed into a behavioral driver. This transformation chain of “social normative pressure → internal control beliefs” is clearly empirically represented within our chained framework.

This internalization process can be understood through the lenses of relationship need satisfaction in Ryan and Deci’s Self-Determination Theory and Bandura’s vicarious efficacy construction (45–47). During the psychologically fragile period following a sports injury, an individual’s own sense of efficacy temporarily collapses due to the traumatic experience. The companionship and validation from coaches, teammates, and family serve as external compensatory efficacy, providing the individual with a psychological secure base to attempt recovery (45). As rehabilitation continues, the successful experiences generated by external support are gradually internalized into independent self-efficacy, achieving the transition from “external lifting” to “internal standing” (48). In empirical studies of exercise rehabilitation adherence, social support has been confirmed as a key factor predicting rehabilitation adherence (49). Levy et al., in a longitudinal study of 70 injured athletes, empirically verified this relationship relatively early; their findings are consistent with ours, showing that the promotive effect of social support on rehabilitation adherence is primarily achieved by enhancing the individual’s internal sense of mastery, rather than by applying external normative pressure (50). This collectively points to a practically significant conclusion: in social support interventions for sports rehabilitation, providing purely instrumental support is insufficient. It is crucial for significant others to convey the message “you have the ability to do this” through encouragement, rather than merely exerting the normative pressure of “you should exercise”.

### 5.5 Research limitations and future directions

First and foremost, the cross-sectional design fundamentally precludes causal inferences. Although our hypothesized pathways were grounded in strong theoretical temporal ordering (HBM → TPB → Intention), the mediation effects revealed in this study are essentially statistical decompositions of contemporaneous covariances rather than empirical validations of causal sequences. For instance, the pathway from attitude to control beliefs may also involve reverse feedback (i.e., high control beliefs, in turn, reinforce positive attitudes)—a reciprocal dynamic that cross-sectional data cannot disentangle. Future research should employ multi-time-point measurements (e.g., acute phase, functional recovery phase, return-to-sport phase) combined with cross-lagged panel models to test the dynamic causal relationships among the variables.

Second, the sample was derived from a single normal university, resulting in a disproportionately high female ratio (69.4%), and caution is required when generalizing these findings to other types of institutions and male-dominated populations. Although existing epidemiological data indicate that the overall incidence rates of sports injuries among Chinese university students do not differ substantially by gender—26.2% for males versus 23.2% for females (1)—injury types (e.g., males are more prone to acute sprains, whereas females are more susceptible to patellar overuse injuries) and psychological coping patterns may vary across genders. Women’s tendencies toward higher pain sensitivity, elevated anxiety levels, and greater reliance on social support may result in differential pathway strength in our model—for instance, the “perceived severity → attitude suppression” path or the “subjective norm → control beliefs” path might exhibit distinct patterns in male populations. The predominance of non-sports majors (83.1%) further implies that our sample may reflect a general student population rather than high-performance athletes, who might exhibit stronger risk-taking tendencies, higher pain tolerance, or different coping strategies (e.g., greater reliance on coach feedback). Thus, our model may be more representative of recreational exercisers than competitive athletes. Future studies should conduct multi-center, cross-institutional comparative research and systematically test whether gender moderates the model pathways, and intentionally stratify samples by academic major and athletic involvement to test the invariance of our model across these subgroups.

Additionally, one of the perceived severity items—“I am afraid of being injured again while exercising, and this worry often comes to mind”—may partially overlap with fear-avoidance cognition, which could have strengthened the observed negative effect of perceived severity on attitude to some extent. Future research could adopt more pure measures of severity perception (e.g., items focusing solely on the objective assessment of injury consequences) to further clarify the relationship between perceived severity and fear-avoidance beliefs.

## 6 Conclusion

By integrating the HBM and the TPB, this study constructed a chained mediation model of university students’ continuous exercise intention following a sports injury. The study yields three primary findings. First, the transformation of health beliefs into intention exhibits a dual-route mechanism. Perceived benefits were associated with intention via a chained pathway of attitude → control beliefs; conversely, perceived susceptibility and barriers were indirectly related to lower control beliefs, and this relationship did not operate through attitude. Second, perceived severity exhibited a significant negative suppression of rehabilitation attitude, a pattern consistent with the fear-avoidance model, suggesting that excessive threat perception may activate avoidance responses in the injured party. Finally, the contextually reconstructed construct of control beliefs regarding exercise rehabilitation served as the core hub of the model, effectively internalizing value cognition and external subjective norms, and showing the largest predictive effect size on behavioral intention in the present model.

Based on these findings, we outline the following candidate directions for future intervention design and empirical testing:

First, de-escalation guidance with benefit-oriented empowerment may be a promising strategy. Rather than repeatedly exposing students to alarming images or case examples of reinjury consequences—which may inadvertently heighten fear-avoidance—health education campaigns could adopt “successful return-to-sport narratives” and “progressive benefit visualization” (e.g., weekly rehabilitation milestone check-ins that visually track functional gains). This cognitive reframing would shift students’ focus from “avoiding negative outcomes” to “attaining positive gains.” For example, rehabilitation staff could implement a 5-min weekly “benefit reflection” exercise, asking students to list three functional improvements they have noticed (e.g., “I can now bend my knee 10 degrees further than last week” or “I feel more stable when walking”), thereby reinforcing perceived health benefits and strengthening the cognitive foundation for sustained exercise intention.

Second, the reconstruction of control beliefs appears to be a core intervention focal point. Intervention may need to go beyond knowledge delivery to provide individualized “minimal success experience” rehabilitation prescriptions—for example, progressing from weight-bearing-free joint mobility exercises to gradually intensified resistance training—so that students gain tangible mastery at each stage. Supplementing this with an accessible online Q&A channel for rehabilitation concerns could further dismantle practical execution barriers and sustain perceived competence. A practical implementation could involve designing a structured 12-week graded activity schedule, with daily logs for students to record their perceived confidence levels before and after each session, allowing both students and therapists to track mastery experiences and calibrate progression difficulty in real time.

Third, an empowering social support network might facilitate rehabilitation adherence. Training coaches and peers in supportive communication scripts—for instance, reframing “you should go to rehabilitation” into “I saw you did really well last time; let’s give it another try”—could enable external encouragement to function as a source of vicarious efficacy. This external support, when consistently delivered, may be gradually internalized into the student’s own autonomous confidence to persist in rehabilitation exercise. To operationalize this, universities could establish a “peer rehabilitation partner” system, pairing injured students with trained peer supporters who provide weekly encouragement calls, share their own recovery experiences, and model positive coping strategies. This relational scaffolding can be particularly potent during the psychologically vulnerable early rehabilitation phase, when students’ own self-efficacy is most fragile. These candidate components await validation in future experimental or intervention studies.

## Data Availability

The original contributions presented in the study are included in the article/Supplementary material, further inquiries can be directed to the corresponding author/s.
